# Benefits and resource implications of family meetings for hospitalized palliative care patients: research protocol

**DOI:** 10.1186/s12904-015-0071-6

**Published:** 2015-12-10

**Authors:** Peter L. Hudson, Afaf Girgis, Geoffrey K. Mitchell, Jenny Philip, Deborah Parker, David Currow, Danny Liew, Kristina Thomas, Brian Le, Juli Moran, Caroline Brand

**Affiliations:** 1 Centre for Palliative Care St Vincent’s Hospital, University of Melbourne, Melbourne, Australia; 2 Queens University, Belfast, UK; 3 Centre for Oncology Education and Research Translation (CONCERT), Ingham Institute for Applied Medical Research, South Western Sydney Clinical School, UNSW Medicine, The University of New South Wales, Sydney, Australia; 4 School of Medicine, University of Queensland, Queensland, Australia; 5 Centre for Applied Nursing Research, Ingham Institute for Applied Medical Research, School of Nursing and Midwifery, Western Sydney University, Sydney, Australia; 6 Discipline, Palliative and Supportive Services, Flinders University, Adelaide, South Australia; 7 Melbourne EpiCentre, University of Melbourne and Melbourne Health, Melbourne, Australia; 8 Palliative & Supportive Care Melbourne Health & University of Melbourne, Melbourne, Australia; 9 Palliative Care Austin Health, Melbourne, Australia; 10 Department of Epidemiology and Preventive Medicine, Monash University Melbourne, Melbourne, Australia

**Keywords:** Palliative, Family meeting, Family conference, Family carers, Family caregivers, Intervention, Trial, Health economics, Quality of life, Unmet need

## Abstract

**Background:**

Palliative care focuses on supporting patients diagnosed with advanced, incurable disease; it is ‘family centered’, with the patient and their family (the unit of care) being core to all its endeavours. However, approximately 30–50 % of carers experience psychological distress which is typically under recognised and consequently not addressed. Family meetings (FM) are recommended as a means whereby health professionals, together with family carers and patients discuss psychosocial issues and plan care; however there is minimal empirical research to determine the net effect of these meetings and the resources required to implement them systematically.

The aims of this study were to evaluate**:** (1) if family carers of hospitalised patients with advanced disease (referred to a specialist palliative care in-patient setting or palliative care consultancy service) who receive a FM report significantly lower psychological distress (primary outcome), fewer unmet needs, increased quality of life and feel more prepared for the caregiving role; (2) if patients who receive the FM experience appropriate quality of end-of-life care, as demonstrated by fewer hospital admissions, fewer emergency department presentations, fewer intensive care unit hours, less chemotherapy treatment (in last 30 days of life), and higher likelihood of death in the place of their choice and access to supportive care services; (3) the optimal time point to deliver FM and; (4) to determine the cost-benefit and resource implications of implementing FM meetings into routine practice.

**Methods:**

Cluster type trial design with two way randomization for aims 1-3 and health economic modeling and qualitative interviews with health for professionals for aim 4.

**Discussion:**

The research will determine whether FMs have positive practical and psychological impacts on the family, impacts on health service usage, and financial benefits to the health care sector. This study will also provide clear guidance on appropriate timing in the disease/care trajectory to provide a family meeting.

**Trial registration:**

Australian New Zealand Clinical Trials Registry ACTRN12615000200583.

## Background

Acute hospitals provide end-of-life care to the majority of people who die in Australia and many other countries [1]. Palliative care focuses on supporting patients diagnosed with advanced, incurable disease. It is ‘family centered’, with the patient and their family considered to be the unit of care [2]. Given the significant burden associated with caring for a dying relative, the World Health Organisation (WHO) advocates that health care services focus on enhancing family members’ quality of life [3]. Accordingly, many nations have established standards and policies for responding to the needs of family carers as well as patients [4–6]. Despite these initiatives, patients with advanced disease may be exposed to aggressive or futile treatment, and approximately 50 % of carers experience psychological distress, which is typically under-recognised [7].

Communication with patients and their family is a vital element of palliative care. Information exchange, preparing for discharge and assessing needs is recommended standard practice. Only when carers of patients with advanced disease are well informed are they able to provide high quality end-of-life care for their relative [8]. Inadequate communication can have profound negative effects resulting in psychological distress due to unmet information needs, lack of shared decision making and mistrust of healthcare providers [9]. Open discussions between health professionals and family carers are an effective way of providing psychological support [10]. However, the quality of communication can vary considerably and communication failures are the most common reason for complaints in health care, with end-of-life care being no exception [1]. Interventions with family carers which focus on responding to needs and preparing them for their role have produced demonstrable improvements in carer well-being and sense of preparedness [11, 12], reduction of unmet needs [11, 13–16], and carer burden and increased quality of life and knowledge of patient symptoms [17]. Findings from systematic reviews also demonstrate that structured information from health professionals can reduce anxiety [18]. The benefits of family carer involvement in discharge planning have also been reported [19].

One of the most important clinical tools for healthcare providers to facilitate communication for people with advanced disease is a family meeting [20–22]. Family meetings (also known as family conferences), are meetings between the family carers, the patient (where possible) and health care professionals, and are undertaken for multiple purposes including psychosocial support, clarifying the goals of care, discussing diagnosis, treatment, prognosis, discharge planning and developing a plan of care for the patient and carer [23]. Studies on family meetings in intensive care units (ICUs) have demonstrated improvement in communication [20, 24], decrease in carer burden [25], and, importantly, reduction in length of stay [26]. Family meetings in the ICU have also led to measurable benefits including decreased post-traumatic stress disorder, anxiety and depression [20, 27].

Family meetings are recommended as a core intervention within the context of palliative care provision [28]. These encounters, however, are not usually provided consistently or systematically, nor are they conducted according to best available evidence [29]. Moreover, the specific outcomes for patients, family carers and health professionals associated with conducing a family meeting within the palliative care setting are underexplored.

### Structured family meetings guidelines and pilot work

Hudson and colleagues developed clinical practice guidelines [30] for conducting Structured Family Meetings (SFMs) for those patients referred to specialist palliative care in hospitals.

The meetings are intended to take no longer than one hour and should be conducted by health care professionals with relevant training in facilitating such meetings. Hudson and colleagues subsequently examined the feasibility and effectiveness of the SFM clinical guidelines in an observational study using mixed methods evaluation [31]. The following outcomes were demonstrated: (1) family carers reported significant reductions in unmet needs; (2) family carers reported that meetings were informative and useful; (3) health professionals reported that the meetings were well-facilitated and (4) family meeting facilitators (provided with specific training to convene and conduct a family meeting) reported that they were better equipped to facilitate the meetings. This study provided preliminary data on the feasibility and acceptability of family meetings as a means of identifying and addressing family concerns. These findings require further testing in a larger sample, using more robust research methods [31]. In addition, it would be advantageous to explore patient outcomes, ascertain any sustained longer term benefits of SFM for family carers (focusing on distress) and discern the best time (within the advanced disease trajectory) to conduct family meetings.

In summary, whilst current guidelines advocate family meetings be routinely conducted for all patients [28], these encounters are typically neither consistently provided nor conducted according to best available evidence. There is therefore justification to further investigate, within a robust research evaluative study design, the impacts and outcomes of SFM for patients, carers and families and the barriers and enablers that may influence effective implementation. Given the major financial and resource implications of SFM, we will also provide clear guidance on the appropriate timing in the disease/care trajectory to provide a family meeting.

The purpose of this project is to evaluate outcomes for patients and family carers who attend a SFM and determine the cost and resource implications of implementing SFM into standard practice for hospitalised patients with advanced disease.

Our aims are:

1. To assess the short and longer term effect of SFM on patient and family carer outcomes and determine the most appropriate time point in a patient’s advanced disease trajectory to provide a SFM.
2. To determine the cost-benefit and resource implications of implementing SFM meetings into routine practice.

## Methods

The following sections detail the methods for addressing each of the project aims.

### Aim 1

To assess the short and longer term effect of SFM on patient and family outcomes and determine the most appropriate time point in a patient’s disease trajectory to provide a SFM.

Our alternative hypotheses for Aim 1 are:

- (H1) Family carers of hospitalised patients with advanced disease (referred to a specialist palliative care in-patient setting or palliative care consultancy service) who receive a SFM will report significantly lower psychological distress (primary outcome), fewer unmet needs, increased quality of life and feeling more prepared for the caregiving role.
- (H2) Patients who receive the SFM will experience appropriate quality of end-of-life care, as demonstrated by fewer hospital admissions, fewer emergency department (ED) presentations, fewer intensive care unit hours, less chemotherapy treatment (in last 30 days of life), and higher likelihood of death in the place of the person’s choice (in consultation with their caregivers) and access to supportive care services.
- (H3) Patients and family carers who receive a SFM earlier in their advanced disease trajectory (i.e further way from death) will have more favourable outcomes (as per outcomes listed in H1 and H2).

#### Research design

This study will randomise three participating sites: all sites will collect baseline data (i.e. Time 1, 2 & 3 data) for 6 months; then two of the three sites will deliver the intervention. As such, one site will remain the control site for the entire time and there will be before and after measurements (with excellent baseline data collected prospectively) for the two sites which will participate in the intervention. As each site will have baseline data, any differences between sites can be identified and, if necessary, controlled for during the analyses. The intervention recruitment period is longer than the control period in the two intervention sites to ensure that the total numbers of control and intervention participants are approximately equivalent). This design was deemed the most appropriate and feasible method for this type of health service research, since randomisation by patient in each service risks cross-group contamination within that clinical setting. A conventional cluster trial or stepped-wedge design is not feasible due to the significant number of sites that would be required. Sites will be randomised by an independent statistician via a random number generation system.

#### Participants

##### Inclusion criteria for patients

Hospitalised patients with advanced, non-curable disease referred to a specialist palliative care in-patient unit or palliative care consultancy service for inpatients, who have a person (relative or friend) whom they perceive to be their main support person (i.e primary family carer), who are willing for the research team to approach the family carer for potential research participation and agreeable to have their medical information shared with the family carer, if pertinent, during the SFM. *Exclusion criteria for patients:* Under 18; likely to die within 1 week (as assessed by treating physician or senior nurse); unable to identify a primary family carer, unable to provide informed consent due to cognitive impairment or incapacity to comprehend and speak English (as assessed by treating physician, senior nurse or research assistant).

##### Inclusion criteria for family carers

Nominated by the patient as their primary support person and willing to attend a SFM if allocated to the intervention arm.

##### Exclusion criteria for family carers

Under 18; incapacity to understand or speak English; unable to provide informed consent due to cognitive impairment (as assessed by the research assistant); or not agreeable to being considered the primary family carer.

For the intervention phase, patients and family carers who have already been involved in a formal family meeting as part of routine specialist palliative care will be excluded from participation. As part of routine data collection for the study we will ask each participating site to record in the medical record if a formal family meeting (ie specific meeting set up to confirm plan of care) has occurred.

#### Procedure

**Fig. 1:**
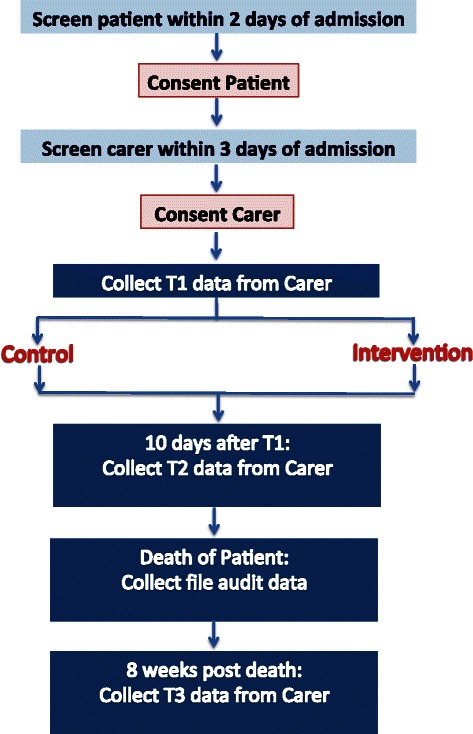
Recruitment and data collection

Recruitment (see Fig. 1) will occur as soon as possible (within 48 h) after the patient is admitted to the palliative care unit or seen by the consultancy service. The research assistant will screen the list of new admissions to both the inpatient unit and the consultative team daily and speak with the clinical team to check eligibility. They will approach eligible patients in person within 48 h of admission to give patients a copy of the Plain Language Statement and consent form and invite patients to participate. Patients will need to consent to their medical records being accessed and their medical details being discussed at the family meeting (if well enough and if allocated to the intervention group). Patients will need to identify a family carer (as per inclusion criteria) and provide consent for the research assistant to contact them about the study. Patients will not be required to provide any self-report data.

The research assistant will then phone the family carer (or approach them in person at the hospital) to invite them to participate within 72 h of patient admission (see Fig. 1). The research assistant will give the family carer a copy of the Plain Language Statement and consent form if approaching them in person and go through it with them and obtain formal written consent. If contact has been made by phone, the research assistant will go through the content of the Plain Language Statement and consent form with them over them phone and send it to them (either by email or post) to be signed and returned by post or in person. Once the carer has consented, the research assistant will arrange a convenient time (and place) to collect time 1 data either over the phone or in person (at the hospital). Time 1 data collection will take no longer than 30 min to complete and will be conducted within 1 week from referral/admission.

The research assistant will phone the family carer when it is time to collect time 2 and 3 data and ask them if they would prefer to complete the questionnaire over the phone, in person at the hospital, or by post. Times 2 and 3 data collection will take no longer than 30 min to complete.

If the family carer is in the intervention group, the research assistant will refer them to the family meeting facilitator (who has been trained to conduct the structured family meetings) as soon as the carer has completed time1 data. Similarly, once the family meeting has been conducted (ideally within a week of time 1 data being collected) the family meeting facilitator will inform the research assistant. This study design does not allow the research assistant to be blinded to the condition to which carers are allocated.

If the family carer is in the control group, then they receive usual care for the period between time 1 and time 2 data collection.

#### Standard care

Standard care is known to be quite heterogeneous. Family meetings are offered in all participating sites but the frequency and method of conduct varies; thus we are mindful that some control participants will receive a family meeting, although these are typically not formally structured. Our research has been designed in recognition of this and we will also collect data on the frequency of family meetings conducted in the control group.

#### Intervention

The intervention which is administered according to clinical guidelines for conducting family meetings is comprehensively outlined in the earlier work of Hudson and colleagues [32]. In summary, the guidelines incorporate: (1) principles for conducting family meetings; (2) pre-meeting procedures such as liaising with the patient/family and prioritising issues; (3) deciding who needs to attend the family meeting; (4) a procedure for conducting the meeting; and (5) strategies for follow up after the meeting, including data on whether or not the priority issues for the family carer have been addressed. The meetings are intended to take no longer than one hour.

Approximately four facilitators will be appointed at each of the participating sites to convene and conduct the family meeting/intervention. The facilitators will be nurses, doctors, social workers or psychologists with extensive clinical palliative care experience and relevant academic qualifications. Facilitators will be identified by the director of palliative care at each of the participating sites. They will receive training in administering the intervention which will incorporate conducting the family meeting according to the aforementioned guidelines; along with communications skills theory and practicum. In order to limit variation of intervention delivery at each site the training approach for all facilitators will be consistent and will be conducted face to face over the course of 1 day.

#### Data collection

Eligibility data will be recorded along with reasons for refusal to participate (for those eligible who are willing to provide this information). Data collection will involve mixed methods including quantitative and qualitative data.

##### Family carer measures

We have administered similar quantity and type of measures to family caregivers with no negative sequelae apparent [33].

1. The 12 item version of General Health Questionnaire (GHQ) [34]. The GHQ has demonstrated good reliability and validity and is commonly used as a screening tool to detect those likely to develop psychiatric disorders. It is a measure of the common mental health problems including the domains of depression, anxiety, somatic symptoms and social withdrawal [35, 36]. The GHQ is scored on a Likert scale, with a maximum score of 36; score ≥15 indicates evidence of distress; and score >20 suggests severe problems and psychological distress. Family carers who meet the cut off for profound psychological distress (>20) at T1 will be advised of such and recommended that they seek formal medical/psychological review from their treating doctor or palliative care team. They will however still be eligible to participate in the study. GHQ will be administered at all three time points.
2. The Family Inventory of Needs (FIN) [37] has 20 items and has been shown to have good reliability and validity [37, 38] . Administered at times 1 and 2 only as this focuses on caregiving while the patient is alive.
3. The Preparedness for Caregiving Scale (PCS) [39] has 8 items and has been shown to have good reliability and validity [38] . Administered at times 1 and 2 only as this measure focuses on caregiving while the patient is alive.
4. The Short form health survey version 2 (SF12-v2) is 12 items quality of life measure that has been widely used across many diverse populations. It is a measure of both physical and emotional quality of life [40]. Administered at all three time points.
5. The Caregiver Quality of Life Index-Cancer (CQOLC) scale is a measure of caregiver quality of life. The scale has been found to be a reliable and valid measure of quality of life for caregivers of cancer patients and cancer patients receiving palliative care [41]. As the questions do not specifically relate to cancer, they are appropriate for caregivers of non-cancer palliative care patients. Administered at times 1 and 2 only as this measure focuses on caregiving QOL while the patient is alive.
6. Family carers will also complete a socio-demographic survey including details and level of function of their relative via the Australian Karnofsky scale [42]. Administered at T1 only.
7. At time 3 (after death) the family carer will also complete a measure of the quality of the death which will be measured using a shortened version of the Quality of Death and Dying Questionnaire [43, 44]. This measure has been widely used with similar populations and includes 17 items that measure different aspects of the dying experience.

We will also collect data on the date of patients’ death to assist with determining the impact of having the family meeting further away from death.

##### File audit (Patient record)

For the second hypothesis (H2), data will be collected from the patients’ medical records using a File Audit Checklist and also from data from the hospital administrative data sets (which include ICD-10-AM codes which are collected for the Department of Health). These data will include indicators of quality end-of-life care [45]: hospital admissions and length of stay, ED presentations, hours in ICU, chemotherapy administration in the last 30 and 14 days of life, a new regimen of chemotherapy in last 30 days of life, and place of death. Data will also be collected on access to supportive care services, evidence of advanced care plans, and attendance at family meetings.

#### Sample size, power calculation and feasibility

The primary outcome for H1 is measured via the GHQ, a 12-item scale with a possible range of 0-36. Our previous study [46] of 275 family carers in which the mean GHQ difference was 2.8 (SD = 5.6), represents a medium effect size (ES) of 0.5 which is a common estimate of a minimal important difference [47]. The sample size calculations are based on a smaller mean difference between groups of 2.4, as some carers in the control group will have family meetings as part of standard care. Estimated sample size for two-sample comparison of means Test Ho: m1 = m2, where m1 is the mean in population 1and m2 is the mean in population 2 is 97 per group (total *n* = 194). This calculation assumes that alpha = .05 (two sided), power = .90 (90 %), m1 = 16.8, m2-14, sd1 = 6, sd2 = 6, n2/n1 = 1.00. This sample size allows for sub-group analyses of the consult service compared to the inpatient service or other similar sub-group analysis.

For the three recruitment sites, we have approximately 1900 palliative care inpatient admissions per year and 3000 palliative care consult service admissions per year. However, many consult service admissions become inpatient admissions. Approximately a third of the total admissions per year would be both consult admissions and inpatient admissions, leaving 3280 admissions per year that reflect separate individuals. Based on eligibility rates and response rates of similar previous studies we can assume 50 % of these are eligible (*n* = 1640), ineligibility mostly due to being imminently dying. A further 33 % of those who are eligible consent to participate (*n* = 546). Of the 546 that consent, we assume that 60 % will complete data collection at Time 1 (*n* = 327). Assuming 30 % attrition at T2 (leaving *n* = 228) and 50 % attrition at T3 (since it is during bereavement, attrition may be quite high), 114 cases can be completed per year, providing the required sample size of 228 from the recruitment pool in 2 years.

#### Ethical considerations

Ethical clearance has been obtained at each of the participating hospitals: St Vincent’s Hospital Melbourne, Austin Health, Melbourne Health - Australia. The trial has also been registered with the Australian New Zealand Clinical Trials Group ACTRN12615000200583.

#### Analyses plan

The main analyses will be performed on pooled data across sites for the intervention and control groups, following an analysis of the baseline data to identify any differences between sites. We will undertake analyses as follows. Summary statistics will be calculated to compare the characteristics of carers in each group. Linear mixed models will be used to (1) compare patterns of change over time by testing the intervention group by time interaction and (2) estimating and testing differences in scores between groups at T2 and at T3 via linear contrasts, and accounting for the non-independent nature of the data in clusters. Mixed models yield unbiased estimates for data which are missing [48], and is relevant for cluster trials [49]. Sub analyses will be undertaken dependant upon obtaining a suitable sample size. In the event of ‘no intervention effect’, where possible, a sub-analysis will be conducted to compare outcomes in the control group carers who did not have a meeting (i.e. a ‘per protocol’ analysis) with those in the intervention group carers who had a meeting. In the event of ‘no intervention effect’ , a sub-analysis will be conducted on those that were moderately or highly distressed at time 1 to compare outcomes in the control group and intervention group for distressed carers. A sub analysis will also be undertaken to explore any differences in outcomes between participants recruited from the specialist palliative care in-patient facility and those recruited from a palliative care hospital consultancy service.

### Aim 2

To determine the cost-benefit and resource implications of implementing SFM meetings into routine practice.

Health economic modeling will be applied to estimate the cost-benefit of SFM. Decision analysis [50] will be used to compare the downstream consequences of SFM versus standard of care, by extrapolation of efficacy data from the trial. The incorporation of Markov [51] and life-tabling [52] techniques will allow for the modeling of outcomes beyond the duration of the trial. The key output in cost-benefit analyses is net costs, comprising the costs of SFM minus costs saved from the reduction in downstream health services utilisation. Only direct healthcare costs will be measured, and from the perspective of the Australian healthcare system. These will predominantly be costs of inpatient and outpatient hospital care, medications and community-based healthcare services. All health economic analyses will be undertaken in accordance with recommended approaches, such as 5 % discounting of estimated future costs and health gains. To account for any uncertainty in the data, inputs for health economic modeling, sensitivity and uncertainty analyses will be undertaken via Monte Carlo simulation [53].

We will also conduct a process evaluation to determine the resource requirements of administering the SFM. Such data will include the mean time to conduct the intervention, the number and the types of health disciplines involved in the family meeting and whether or not the patient also participated. We will also explore the administrative time involved in setting up and facilitating the SFM.

At the completion of the intervention phase two focus groups will be conducted with health professionals and interviews (approx. 20) with family carers from the intervention sites will be undertaken to explore the perceived impacts and benefits of the SFMs. They will also be used to discern external and internal policy changes; clinician (staff resource, beliefs and attitudes), patient and carer factors (access, timeliness, understanding, information). This will assist with monitoring temporal issues relevant to the study.

All health professionals at each of the two intervention sites who have attended at least two of the SFMs will be invited to participate in a focus group (via a personalised email). The Plain Language Statement and consent form will be attached to the email. At the beginning of the focus group, the project officer will go through the Plain Language Statement; which will provide all potential participants with the assurance that their involvement is entirely voluntary and their participation or otherwise will in no way hinder their employment. Health professionals will be asked to complete the consent form. Once informed consent is obtained from the palliative care health professionals, the project officer will conduct a focus group. The focus group will be taped and transcribed and any identifying information deleted from the transcripts. The focus group will take about 30–60 min.

One in six family carers who complete the intervention will be invited to participate in an interview after the completion of their time 2 questionnaires via a question on the questionnaire with a Yes/No response format and an open-ended space which requests a phone number and best time to call. The research assistant will then call the family carer at the suggested time and ask the carer whether they are still interested in participating in an interview and if they are, they will organise a convenient time. Interviews may be conducted at the hospital or over the telephone. Interviews will be recorded and transcribed. A semi structured interview guide has been developed by the project team and incorporates questions related to: perceptions of the way the family meeting was convened and conducted; reflections on any positive or negative outcomes and recommendations for improving family meetings. It is expected that the interview take approximately 30 min.

Focus groups will be recorded and transcribed and analysed for impacts, benefits and additional content. Interviews will be recorded digitally verbatim and transcribed. NVivo software will be used to assist with storage and coding. The interviews will be analysed using the five steps of thematic analysis recommended by Boyatzi [54] (1) reducing the raw information, (2) creating a code, (3) determining the reliability of the code, (4) identifying themes within subthemes and (5) comparing themes across subsamples. The first two interviews (20 %) from each site will be coded by two members of the research team. A Kappa value of 0.6–0.8 indicating substantial agreement between raters [55] will be achieved at step 3 prior to the research assistants completing steps 4 and 5 for the remainder of the interviews.

#### Study fidelity

One in six intervention meetings at each site will be selected for transcription and analysis to check fidelity of the intervention delivery method; and we will also record information about the attendees and topics discussed and later (a subsequent study) analyse the recordings with respect to a range of factors including the process of communication between the carer, patient and health professionals. Understanding the content and dynamics of family meetings will further inform implementation.

## Discussion

The WHO advocates that palliative care should not only improve the quality of life for patients but also for their families [3]. The principle of family centred care and the inclusion of family carer satisfaction with end-of-life health care is advocated as a key indicator of hospital performance [56]. Current guidelines advocate family meetings to be routinely conducted for all patients [28] which has major financial and resource implications. These encounters however are typically not consistently provided nor are they conducted according to best available evidence. The research described herewith will determine whether SFMs have positive practical and psychological on the family, impacts on health service usage, and financial benefits to the health care sector. This study will also provide clear guidance on appropriate timing in the disease/care trajectory to provide a family meeting.
